# Using AI to Design and Develop Online Educational Modules to Enhance Lung Cancer Screening Uptake Among High-Risk Individuals

**DOI:** 10.3390/cancers18040544

**Published:** 2026-02-07

**Authors:** Fang Lei, Hua Zhao, Feifei Huang, Edris Farhadi

**Affiliations:** 1 School of Nursing, University of Minnesota, 308 Harvard St SE, Minneapolis, MN 55455, USA; 2 College of Nursing, Shanxi University of Chinese Medicine, Jinzhong 030609, China; 3 School of Nursing, Fujian Medical University, No. 1 Xueyuan Road, Minhou County, Fuzhou 350122, China

**Keywords:** educational intervention, Health Belief Model, health promotion, lung cancer screening, online learning, smokers

## Abstract

**Background:** Despite clear evidence supporting low-dose computed tomography (LDCT) for lung cancer screening, the participation rate among eligible high-risk individuals remains low. Educational interventions that address gaps in knowledge, attitude, and beliefs may improve screening uptake. **Objective:** This study describes the systematic use of artificial intelligence to design and develop a series of online educational modules aimed at improving knowledge, attitudes, and beliefs toward lung cancer screening among high-risk individuals. **Methods:** Guided by the Health Belief Model and principles of digital health education, five interactive online modules were developed by artificial intelligence technology to address key topics: (1) lung cancer epidemiology, etiology, signs, and symptoms; (2) lung cancer treatment and care; (3) lung cancer prevention methods; (4) screening guidelines, benefits, and risks; and (5) screening procedures and results interpretation. The design process included literature review, individual cognitive interviews, expert consultation, and pilot testing among target users. Qualitative individual interviews were conducted with 12 high-risk individuals. Content validity was evaluated by an expert panel (*n* = 7) using a content validity index (CVI), and pilot usability testing was conducted with 25 high-risk individuals. **Results:** All five modules achieved high content validity (I-CVI range = 0.90–1.00; S-CVI = 0.96). Usability and satisfaction testing showed that participants rated the modules as clear, engaging, and relevant (mean System Usability Scale score = 88/100, mean satisfaction score = 18.32/20). Participants demonstrated significant improvements in knowledge (*p* < 0.001), lung cancer stigma (*p* < 0.001), and health beliefs (*p* < 0.001) after module completion. Of the 22 participants who completed the 3-month follow-up (88%), 13 (59.1%) reported obtaining LDCT screening. **Conclusions:** The developed online modules demonstrated strong content validity and usability, indicating their feasibility for use in future intervention studies to promote lung cancer screening knowledge, attitude, beliefs, and participation among high-risk individuals.

## Simple Summary

This study describes the development and pilot testing of artificial intelligence-generated online educational modules designed to improve lung cancer screening knowledge, attitudes, and beliefs among high-risk individuals. Guided by the Health Belief Model, five interactive modules were created to address lung cancer risk, prevention, screening guidelines, and screening procedures. Content validity and usability testing demonstrated high expert agreement and strong user satisfaction. Preliminary findings showed significant improvements in knowledge, lung cancer-related stigma, and health beliefs following module completion, with over half of participants reporting completion of low-dose computed tomography screening at the three-month follow-up. These results suggest that AI-assisted, theory-guided digital education is a feasible and promising approach to support lung cancer screening awareness and participation in high-risk populations.

## 1. Introduction

Lung cancer is the leading cause of cancer-related mortality in the United States [1] and is the second most frequently diagnosed malignancy among both men and women [2]. In 2023, approximately 238,000 individuals were newly diagnosed with lung cancer, and more than 127,000 deaths were attributed to the disease [3]. Compared with other common cancers, lung cancer is associated with markedly poorer outcomes, with an overall five-year survival rate of 18.6%, substantially lower than those observed for colorectal, breast, and prostate cancers [4]. Early detection remains limited, as only about 16% of lung cancers are identified at a localized stage, despite a corresponding five-year survival rate of approximately 55% for early-stage lung cancer [4]. Prognosis declines sharply with delayed diagnosis; for individuals diagnosed with advanced-stage (stage IV) lung cancer, the five-year survival rate falls to roughly 4% [4]. Consistent with this pattern, more than half of individuals diagnosed with lung cancer die within one year of diagnosis [4].

Early detection through low-dose computed tomography (LDCT) has been shown to significantly reduce lung cancer mortality; however, screening uptake among eligible individuals remains strikingly low across many settings [5,6]. In the United States, only approximately 5% of eligible high-risk individuals, defined as adults aged 50–80 years with a ≥20 pack-year smoking history who currently smoke or quit within the past 15 years and have no prior lung cancer diagnosis, consistent with U.S. Preventive Services Task Force recommendations [7], undergo annual screening [8]. Similarly low uptake has been reported in other high-income countries where lung cancer screening programs have been introduced or piloted, including the United Kingdom, Canada, Australia, and several European nations, despite differences in healthcare systems and implementation strategies [9,10,11,12,13,14,15,16,17,18,19,20,21]. Across geographic contexts, common barriers to screening include limited awareness, misinformation about benefits and harms, fear of screening results, perceived stigma related to smoking, and poor understanding of eligibility criteria and screening procedures [9,10,11,12,13,14,15,16,17,18,19,20,21].

Despite the proven effectiveness of LDCT, public awareness campaigns and traditional healthcare-based education have had limited reach among high-risk populations. Many high-risk individuals do not receive routine preventive care, face substantial socioeconomic constraints, or lack consistent trust in the healthcare system, making it difficult for conventional approaches to close the screening gap [22]. In addition to structural barriers, significant knowledge gaps and misconceptions, such as the belief that screening is unnecessary in the absence of symptoms or concerns about harmful radiation exposure from LDCT, persist and reduce motivation to participate in screening [23]. Attitude- and belief-related barriers, including lung cancer stigma, self-blame associated with smoking, and fear of diagnosis, further discourage engagement with screening services [23]. Furthermore, existing educational materials often fail to address these interconnected knowledge, belief, and attitudinal barriers in culturally and linguistically appropriate ways, limiting their relevance and effectiveness among diverse groups of high-risk individuals. Many resources do not explicitly confront stigma, normalize screening for asymptomatic individuals, or account for the lived experiences of high-risk individuals from marginalized communities. As lung cancer disproportionately affects individuals from lower socioeconomic backgrounds and medically underserved populations [24], there is a critical need for accessible solutions with the potential to reach individuals beyond traditional clinical settings. Digital interventions may offer one promising avenue to support this goal.

Digital interventions represent a promising, yet underutilized, avenue for expanding reach to high-risk groups. Online educational programs can overcome common logistical barriers by providing flexible, on-demand learning opportunities that individuals can access privately and at their own pace. Prior research shows that digital health education can effectively improve understanding, correct misconceptions, and positively influence health behaviors, particularly among individuals who may avoid in-person clinical encounters due to stigma or fear [25,26]. Moreover, online platforms offer the potential for scalable and cost-effective dissemination, critical components for addressing lung cancer screening disparities at a population level [27]. Digital modules can also be rapidly updated as guidelines evolve, ensuring that learners receive the most accurate and current recommendations.

Recent advances in artificial intelligence (AI) have expanded the toolkit available for developing and scaling digital health interventions in healthcare delivery and preventive screening. Across cancer care, AI has been widely applied in diagnostic and clinical domains, including risk prediction, image interpretation, and clinical decision support (e.g., Qure.ai and Lunit), demonstrating promise in improving efficiency and access to care [28]. In the context of lung cancer screening education, AI-assisted platforms enable efficient content development and scalable dissemination of educational materials beyond traditional healthcare settings, which may help reach high-risk individuals who experience structural barriers, limited healthcare engagement, or distrust of the medical system [29]. While web-based educational tools and interactive modules have been shown to improve knowledge, perceived self-efficacy, and engagement in screening decisions [30,31], most existing AI tools in healthcare focus on symptom triage, clinical decision support, or automated documentation rather than theory-guided educational content (e.g., Lunit). Compared with these contemporary tools, our study advances the field by integrating AI-assisted video and interactive content development with the Health Belief Model to create sequential, theory-based modules tailored specifically for lung cancer screening awareness and motivation. This approach not only leverages scalable AI technologies to generate content efficiently but also ensures that the educational materials align with evidence-based behavioral constructs that are known to influence screening uptake, filling a gap in current AI applications in preventive health education.

To design interventions that meaningfully shift screening behaviors, it is essential to account for the cognitive and psychological determinants that influence decision-making. The Health Belief Model (HBM) provides a strong theoretical foundation for guiding educational content, emphasizing perceived susceptibility, severity, benefits, and barriers, as well as cues to action and self-efficacy [32]. High-risk individuals often underestimate their personal risk or hold fatalistic attitudes about lung cancer; carefully targeted educational content grounded in HBM can directly address these gaps. Incorporating behavioral theory into educational design ensures that the modules go beyond simply providing information and actively support motivational and cognitive processes associated with health behavior change. This theoretical grounding also enhances the likelihood that educational exposure will translate into meaningful, measurable changes in screening intention and behavior.

Given these substantial unmet needs, this study aimed to design, develop, and preliminarily validate five online educational modules addressing key gaps in lung cancer knowledge, screening attitudes, and beliefs among high-risk individuals. Using an evidence-based, theory-driven framework, the study focused on content development, expert validation, usability assessment, and exploratory pilot testing of an AI-supported educational approach that could inform future, rigorously powered studies. By documenting the design and validation process, this study contributes foundational evidence to support subsequent research examining the impact of digital educational interventions on lung cancer screening behaviors.

## 2. Methods

### 2.1. Theoretical Framework

**Figure 1:**
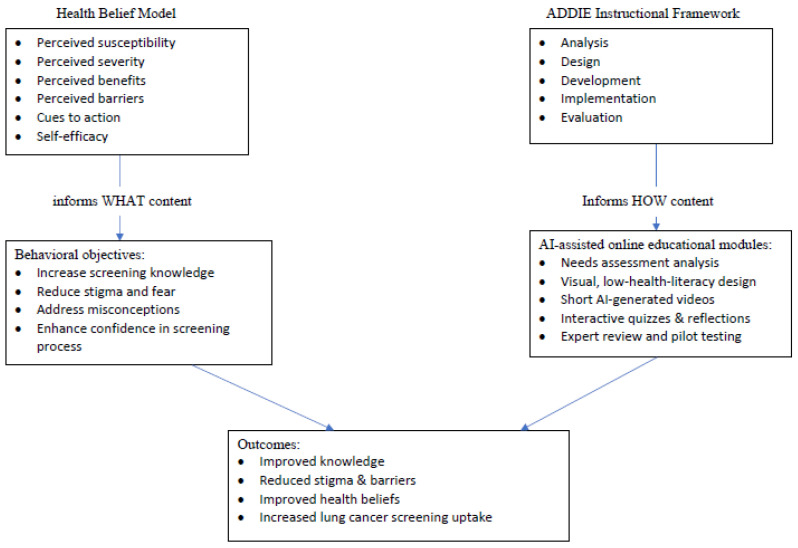
Theoretical Framework of the Study.

The development process was guided by the HBM and the Instructional Design ADDIE framework (Analysis, Design, Development, Implementation, Evaluation) (Figure 1) [33]. The HBM constructs were used to inform educational content, while the ADDIE model structured the design process. The integration of these two frameworks ensured that the modules were both theoretically grounded and systematically developed, enabling alignment between behavioral objectives and instructional strategies. The HBM guided what content should be addressed to influence screening behaviors, whereas ADDIE guided how the content should be designed, delivered, and refined.

### 2.2. Ethical Considerations

This study was conducted in the United States and approved by the University of Minnesota Institutional Review Board (IRB Protocol: STUDY00020835). All study procedures were carried out in accordance with applicable ethical and regulatory standards. Participants were informed of the study purpose, procedures, potential risks, and their right to withdraw at any time without penalty. Data were collected anonymously on REDCap, stored securely on password-protected servers and devices, and analyzed in aggregate to protect participant confidentiality. No identifying information was linked to questionnaire responses, and all procedures adhered to ethical guidelines for research involving human subjects.

### 2.3. Design Process

**Figure 2:**
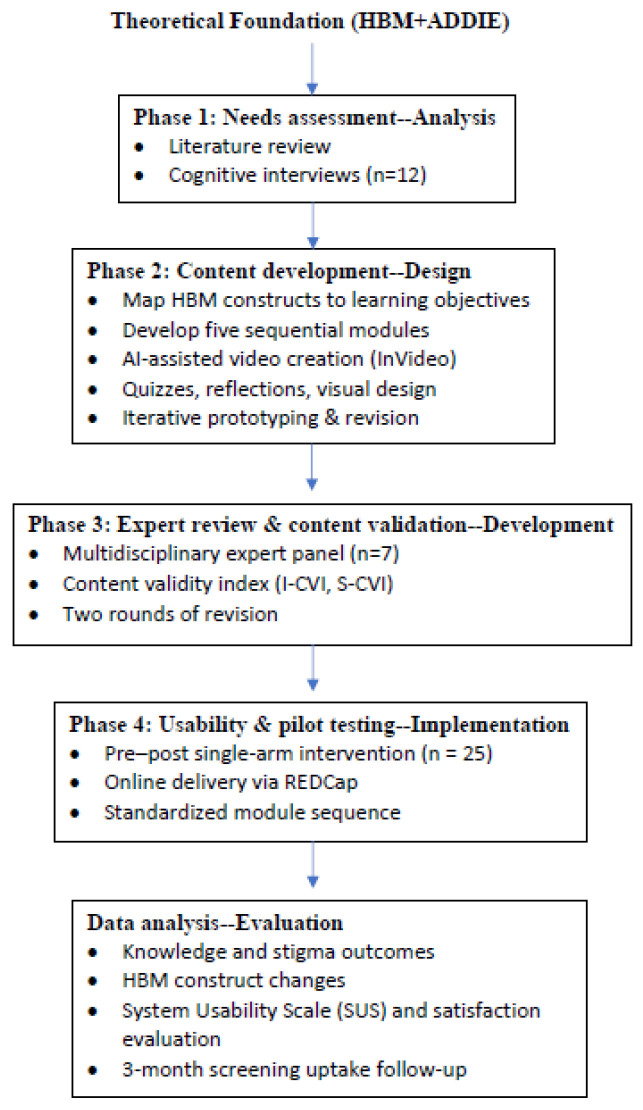
Flowchart of the Design Process.

The design process of the online educational modules is shown in Figure 2, including four phases.

#### 2.3.1. Phase 1: Needs Assessment

Following a preliminary literature review, qualitative individual cognitive interviews were conducted with 12 high-risk individuals (aged 50–80 years, ≥20 pack-year smoking history, current smoker or quit smoking in the past 15 years, without lung cancer or cognitive diseases) via phone or in person, using a semi-structured interview guide [34]. Participants (Mean age = 60, female = 8.33%, married = 75%, and had at least a bachelor’s degree = 75%) identified knowledge gaps, misconceptions, and preferred learning formats. Common themes included: lack of understanding of screening eligibility and procedures; misconceptions about radiation risk and screening cost; fear of being diagnosed with cancer; and desire for credible, easy-to-understand educational materials. Findings informed the selection of topics and the tone of the educational materials. In addition, participants provided input on preferred user interface styles, video length, color schemes, and readability levels. Their feedback helped determine the appropriate health literacy level (6th–8th grade) and informed decisions to incorporate more visual elements, minimize medical jargon, and include testimonials to increase perceived relevance and trust.

#### 2.3.2. Phase 2: Content Development

Integrating findings from the literature review and individual cognitive interviews, five modules were created to sequentially build understanding and motivation for screening (Table 1). Each module incorporated short video lectures (3–5 min) created by the AI-based video production platform InVideo, interactive quizzes and reflection questions to reinforce learning, and a relevant references list to facilitate further understanding of the topics. All AI-generated content was based on evidence from peer-reviewed publications, clinical guidelines, and authoritative health sources, which were explicitly cited in the module reference lists to ensure scientific rigor. Content development occurred between June 2024 and July 2024, during which iterative prototyping was conducted. Draft versions of video scripts, infographics, and interactive elements were evaluated by the study team and revised based on clarity, engagement, and accuracy. The modules were designed to progressively activate HBM constructs—starting with raising awareness of susceptibility and severity, followed by benefits, addressing barriers, and culminating in strengthening self-efficacy and cues to action.

The InVideo platform (https://invideo.io/) was selected because it offered an accessible, scalable, and time-efficient workflow for transforming evidence-based scripts into narrated educational videos without requiring advanced technical expertise or custom software development. At the time of content creation, InVideo employed proprietary, text-to-video generative models that automate voiceover generation and visual animations. The InVideo 2.0 version was used and a plus membership was purchased to enable completion of the tasks. All educational content, including learning objectives, script content, sequencing, and theoretical alignment, was human-authored and theory-driven. AI-assisted features were used solely to support video rendering and rapid iteration, rather than content generation or pedagogical decision-making.

For video development, the study team first drafted evidence-based scripts aligned with module-specific learning objectives and HBM constructs. These scripts were then pasted into InVideo, where AI-assisted features were used to generate narrated videos by combining text-based content with automated voiceovers, visual animations, and on-screen text. Template selection and visual elements were chosen deliberately by the research team to ensure clarity, consistency, and accessibility, while minimizing medical jargon and emphasizing key lung cancer screening messages. The AI-supported workflow enabled rapid iteration of video drafts, allowing the research team to review, refine, and optimize pacing, language, and visual emphasis to enhance comprehension and engagement among high-risk individuals.

#### 2.3.3. Phase 3: Expert Review and Content Validation

A panel of seven experts (three oncology nurses, two pulmonologists, one behavioral scientist, and one health educator, mean age = 59, female = 12%) reviewed all modules for accuracy, clarity, and relevance. Each item was rated on a 4-point Content Validity Scale (1 = not relevant to 4 = highly relevant). The Item-level Content Validity Index (I-CVI) and Scale-level CVI (S-CVI) were calculated. Experts also provided qualitative feedback on cultural appropriateness, clinical relevance, alignment with current screening guidelines, and emotional tone. Suggested revisions, for example, simplifying explanations of false positives and adding a step-by-step visual guide to LDCT, were incorporated. Modules underwent two rounds of revision until all items met an I-CVI ≥ 0.78 and S-CVI ≥ 0.90, indicating strong content validity [35,36].

#### 2.3.4. Phase 4: Usability and Pilot Testing

A pre- and post- single-arm educational intervention study design was used in this phase. Participants enrolled in Phase 4 were distinct from those who participated in Phase 1 (needs assessment), and no individuals were involved in both phases. A total of 27 participants (with a smoking history of more than 20 pack-years, current smokers or quit smoking in the past 15 years, aged between 50 and 80 years old, without lung cancer or other diseases that substantially impact participants’ life expectancy) were recruited online (StudyFinder website, Instagram, Facebook groups) to evaluate module usability and preliminary efficacy. Participants were screened for their eligibility via email, using a seven-item questionnaire. Twenty-five participants completed the intervention, resulting in a retention rate of 92.59%. The mean age of the participants was 60.88 (SD = 5.16) years. Most participants were female (72%), married (72%), and had at least a bachelor’s degree (76%). The majority reported a household annual income between $45,000 and $139,999 (76%). Of the 25 participants, 44% identified as White, 40% as Black or African American, and 16% as Hispanic or Latino. More than half of the participants (56%) were not currently employed, and 36% did not have health insurance. The majority smoked currently (88%), and none had previously undergone lung cancer screening. Only one participant (4%) reported a family history of lung cancer.

Participants completed the questionnaires on REDCap before and after module completion. Instruments included (1) Lung Cancer and Screening Knowledge Questionnaire, which had 12 questions, and the total score ranged from 0 to 12 [37]. Questionnaire items covered epidemiology, screening guidelines, risk factors, and procedural steps; (2) a shortened version of the Cataldo Lung Cancer Stigma Scale, including nine questions related to lung cancer stigma [38] using 4-point Likert-style responses. The total score of the scale ranged from 9 to 36. Reliability scores of the scale ranged from 0.75 to 0.96, which were at the acceptable to excellent level [38]; and (3) the modified Lung Cancer Screening Health Belief Scale, which was composed of 57 items in six sub-scales. The Perceived Severity, Perceived Risk, Perceived Benefits, Perceived Barriers, and Cues to Action sub-scales used 4-point Likert-style responses with items ranging from strongly disagree (=1) to strongly agree (=4). The Self-efficacy sub-scale used 4-point Likert-style responses with items ranging from not at all confident (=1) to very confident (=4). This scale was developed from our preliminary studies based on the original Lung Cancer Screening Health Belief Scale [39], which showed acceptable content validity [40].

The participants completed the System Usability Scale (SUS) and Satisfaction Feedback Questionnaire on REDCap after module completion. The SUS is a short 10-item questionnaire designed to measure the usability of a system [41]. It is a well-designed survey consisting of 5 questions with positive statements and 5 questions with negative statements, with scores ranging from 0 to 100 [41]. Each question is evaluated with a Likert scale ranging from strongly agree to strongly disagree. The current literature evidence suggests that a mean SUS score of 68 is an acceptable benchmark [42]. Participants’ feedback on the intervention was collected by the Satisfaction Feedback Questionnaire. The questionnaire had five items with 4 Likert-style ordinal level responses. The total score of the questionnaire ranged from 5 to 20, with a higher score indicating a higher satisfaction level of the intervention. A total score higher than 15 shows a satisfied attitude toward the online education modules.

To minimize bias, modules were delivered in a standardized sequence on REDCap. Completion time was monitored automatically through the hosting platform’s analytics. Participants were followed over 3 months by email to assess whether they underwent lung cancer screening. Participants were instructed to respond with “yes” if they had undergone the screening and “no” if they had not. Only participants who replied to the follow-up email were included in the analysis of screening uptake.

### 2.4. Data Analysis

Descriptive statistics summarized demographics, CVI scores, usability ratings, satisfaction rate, and screening uptake. Paired t-tests examined pre-post changes in knowledge, attitude, and beliefs of lung cancer screening. All analyses were conducted using SPSS version 31.0.0 (IBM, New York, NY, USA).

## 3. Results

### 3.1. Content Validation

Experts rated all modules as highly relevant and accurate. I-CVI scores ranged from 0.90 to 1.00 across items. The S-CVI (average) was 0.96, indicating excellent overall content validity.

Experts rated all modules as highly relevant and accurate. Across items, final I-CVI values ranged from 0.90 to 1.00, with no items falling below commonly accepted thresholds for adequacy. The S-CVI (average) was 0.96, indicating excellent overall content validity. Minor wording and clarity revisions were made to selected items between review rounds based on expert feedback; however, these revisions did not significantly change I-CVI values, which remained consistently high across rounds.

### 3.2. Preliminary Efficacy

After completing the online educational modules, participants demonstrated large, practically meaningful improvements in key outcomes. Knowledge scores increased significantly (t = −7.28, d = −1.46), while lung cancer stigma scores decreased notably (t = 6.28, d = 1.26). Similarly, large effect sizes were observed across all subscales of the modified Lung Cancer Screening Health Belief Scale, including perceived susceptibility (t = −5.19, d = −1.04), benefits (t = −7.51, d = −1.50), cues to action (t = −8.08, d = −1.62) and self-efficacy (t = −5.79, d = −1.16). In contrast, perceived barriers showed a substantial reduction (t =9.09, d = 1.82), and perceived severity decreased with a moderate effect (t = 4.00, d = 0.80). Collectively, these effect sizes indicate moderate to large magnitude changes, suggesting clinically meaningful shifts despite the pilot sample size. Of the 22 participants who completed the 3-month follow-up (88%), 13 (59.1%) reported obtaining LDCT screening (Table 2).

Participants demonstrated the largest changes in self-efficacy and perceived barriers, suggesting that the modules were effective in increasing confidence in completing the screening process, addressing common concerns, and reducing obstacles to lung cancer screening. In particular, participants reported decreased worries about radiation exposure and fear of diagnosis, two of the most frequently cited deterrents among high-risk individuals. Improvements in perceived benefits were also observed, reflecting increased recognition of the value of early detection and the potential life-saving impact of screening.

### 3.3. Usability Testing and Satisfaction

Participants completed all modules within 90 to 120 min. The mean SUS score was 88, indicating high usability and ease of use. Overall, participants reported high satisfaction with the online intervention program. The mean satisfaction score was 18.32 (SD = 2.33).

## 4. Discussion

This study developed and validated five online educational modules to enhance lung cancer screening knowledge, attitude, beliefs, and uptake among high-risk individuals. Guided by the HBM, the modules addressed cognitive and emotional barriers to screening, improved knowledge, decreased stigma, and increased beliefs and participation in LDCT screening.

The high content validity, usability ratings, and satisfaction rate indicate that the modules are accurate, user-friendly, and engaging for the target audience. The significant improvement in knowledge, attitude, beliefs, and screening uptake following module completion highlights their potential as an effective digital health intervention. These improvements are especially notable given the persistent challenges faced by high-risk individuals, such as fear, fatalistic beliefs, and misunderstanding about LDCT, which often hinder participation in recommended screening [23]. The modules’ ability to shift multiple psychological constructs simultaneously suggests strong internal coherence and effective alignment with HBM-guided belief reframing.

Prior studies have used brief brochures or clinic-based counseling to educate high-risk individuals [43,44], but few have leveraged AI-designed interactive online learning. Our modules extend prior approaches by integrating AI-generated multimedia content and real-world testimonials, features known to increase message credibility and behavioral engagement [45]. By providing information in a self-paced, accessible format, the modules may also help reduce time and resource barriers that limit the reach of clinic-based education, expanding opportunities for engagement beyond traditional healthcare encounters [46].

Our study leveraged AI tools to develop lung cancer screening educational modules, which enhance the intervention’s efficiency, consistency, and learner engagement. AI-assisted platforms were used to generate short educational videos and create visually engaging multimedia content aligned with each HBM construct. Practical lessons from this process include the importance of providing AI tools with clear instructional prompts grounded in behavioral theory, iteratively reviewing AI-generated content for clinical accuracy and cultural sensitivity and combining AI outputs with expert oversight rather than relying on automation alone. Using AI substantially reduced production time and allowed rapid iteration of content while maintaining fidelity to evidence-based screening guidelines [47]. For researchers and clinicians developing online educational modules, AI can serve as a scalable and cost-effective adjunct, particularly when paired with structured frameworks, pilot testing with end users, and rigorous validation processes to ensure accuracy, relevance, and trustworthiness of the educational materials.

After completing the online educational modules, participants demonstrated significant improvements in understanding lung cancer risk, the purpose and eligibility criteria for LDCT screening, and the benefits and potential harms of screening. Importantly, knowledge gains extended beyond factual awareness to correction of prevalent misconceptions, such as the belief that screening is only necessary when symptoms are present or that a lung cancer diagnosis is invariably fatal. For high-risk individuals, particularly those with long-term smoking histories, these misconceptions often reinforce avoidance and delay in preventive care [23]. By presenting evidence-based information in brief, structured modules, the intervention helped participants reframe lung cancer screening as a proactive and potentially life-saving preventive behavior rather than a confirmation of illness. These findings suggest that targeted, theory-driven educational content can meaningfully shift both declarative knowledge and risk appraisal among populations that have historically been difficult to reach through traditional educational strategies.

The substantial reductions in lung cancer stigma observed in this study align with evidence that digital interventions can create a psychologically safe environment for learning, particularly among individuals who may feel judged or marginalized in traditional healthcare settings [48]. Reducing stigma is especially critical for high-risk individuals, as internalized stigma has been shown to discourage help-seeking, reduce trust in healthcare providers, and lower screening adherence [23]. The stigma reduction achieved in this study highlights the importance of empathetic, nonjudgmental messaging embedded throughout the modules.

Because low LDCT utilization persists even among individuals who are aware of screening guidelines [49], interventions must not only inform but also empower. The improvements observed across multiple domains of the HBM demonstrate that a well-designed digital program can influence the psychological determinants of behavior change. These findings reinforce the message that addressing affective and cognitive barriers and benefits concurrently, rather than focusing solely on knowledge, may lead to more meaningful and sustained behavioral intentions. Additionally, the significant gains in self-efficacy suggest that the modules may help bridge the gap between knowledge acquisition and action, a critical step toward improving actual screening uptake. Enhanced self-efficacy may encourage participants to initiate conversations with their healthcare providers, navigate scheduling processes, and overcome logistical barriers that often prevent screening completion.

### 4.1. Limitations

This study has several limitations. First, the small sample size and single-arm design of the pilot phase limit generalizability and preclude causal inference. Larger, adequately powered randomized controlled trials are needed to rigorously evaluate the intervention’s efficacy.

Second, the exploratory analyses involved multiple pre–post comparisons across knowledge, stigma, and several HBM constructs using paired t-tests, without formal adjustment for multiple testing. Consequently, the risk of Type I error is increased, and statistically significant findings should be interpreted with caution.

Third, the reported screening uptake at 3 months was self-reported, assessed only among follow-up responders, and not independently verified through medical records or registry data. Accordingly, this uptake result should be considered preliminary and exploratory.

Finally, although the intervention was designed with health equity considerations in mind, the pilot sample was relatively highly educated and digitally engaged, reflecting recruitment through online channels. Future research should intentionally evaluate the intervention in more structurally disadvantaged populations, including individuals with lower socioeconomic status and limited digital access, to determine its equity impact.

### 4.2. Implications for Practice and Research

The modules can be integrated into clinical workflows, lung cancer screening and smoking cessation programs, or community outreach initiatives. They provide healthcare professionals with an evidence-based, standardized educational resource that can be accessed remotely and repeatedly. These modules may be particularly valuable for clinics facing time constraints or lacking dedicated patient education personnel. Health systems could also incorporate the modules into patient portals or telehealth visits to support shared decision-making for lung cancer screening. Given their strong usability, the modules could feasibly be adapted for mobile delivery, increasing accessibility for underserved communities.

Importantly, the strong usability scores underscore the feasibility of implementing this approach widely, even among older adults who may have limited digital literacy. This supports the growing evidence that digital education can be accessible and impactful across demographic groups when thoughtfully designed and scaled. However, scaling these modules to larger and more diverse populations may present challenges, including variability in digital literacy, language preferences, cultural relevance, and access to reliable technology. Ensuring the fidelity of theory-informed and evidence-based content while allowing for local adaptation will be critical as implementation expands. In addition, large-scale deployment may require attention to technical infrastructure, data privacy protections, and integration with existing electronic health records or screening workflows.

Beyond lung cancer screening, the modular and theory-guided design of this intervention offers opportunities for expansion to other health issues that rely on preventive screening, early detection, or sustained health behavior change. Similar AI-assisted, Health Belief Model-informed modules could be adapted for conditions such as colorectal, breast, or cervical cancer screening, as well as chronic disease prevention areas including cardiovascular risk reduction, vaccination uptake, and smoking cessation. Future studies may explore how this scalable framework can be customized to address condition-specific risk perceptions and barriers while maintaining a consistent evidence-based structure. Moreover, longitudinal research is needed to evaluate whether the observed changes translate into actual LDCT completion and improved adherence over time. Evaluating implementation outcomes, such as reach, sustainability, and equity, will also be essential to inform the successful scale-up of AI-assisted, theory-guided educational interventions across diverse health contexts.

## 5. Conclusions

The AI-developed online educational modules in this study demonstrated strong content validity, preliminary efficacy, and usability in improving lung cancer screening knowledge, attitude, beliefs, and uptake among high-risk individuals. They represent a promising, scalable intervention for promoting screening awareness and early detection of lung cancer. By addressing both informational and psychological barriers, the modules have the potential to meaningfully enhance patient engagement in LDCT screening. Future research should examine their impact on real-world screening completion and explore opportunities for broader implementation across diverse populations. Overall, this study contributes to the growing field of digital health education and highlights an innovative approach for reducing lung cancer disparities through accessible, evidence-based learning tools.

## Figures and Tables

**Table 1:** Content Development for the Modules.

Module	Topic	Content Summary
1	Lung cancer epidemiology, etiology, signs, and symptoms	Overview of global and U.S. statistics; risk factors (smoking, environmental exposures); early signs and symptoms.
2	Lung cancer treatment and care	Description of treatment options (surgery, radiation, chemotherapy, immunotherapy); survivorship and quality of life.
3	Lung cancer prevention methods	Smoking cessation, avoidance of secondhand smoke, healthy lifestyle promotion, and environmental risk reduction.
4	Lung cancer screening guidelines, benefits, and risks	USPSTF 2021 guidelines; benefits of early detection; potential harms such as overdiagnosis and false positives.
5	Screening procedures and results interpretation	Step-by-step explanation of LDCT procedure, preparation, results interpretation, and follow-up recommendations.

**Table 2:** Preliminary efficacy of the educational modules.

Domain	Pre-Intervention (Mean ± SD)	Post-Intervention (Mean ± SD)	*p * Value
Knowledge	3.76 ± 2.26	8.60 ± 2.27	<0.001
Stigma	25.52 ± 4.72	19.16 ± 4.62	<0.001
Perceived susceptibility	9.88 ± 1.72	12.44 ± 2.16	<0.001
Perceived benefits	17.72 ± 3.09	24.12 ± 3.84	<0.001
Cues to action	15.28 ± 3.29	20.84 ± 3.30	<0.001
Self-efficacy	28.36 ± 4.89	35.28 ± 4.45	<0.001
Perceived barriers	55.64 ± 5.50	44.24 ± 6.95	<0.001
Perceived severity	26.72 ± 4.87	21.76 ± 2.86	<0.001

## Data Availability

All data analyzed in this study are available upon request to the first author.
